# Supplementary material for: Paediatric Histoplasmosis 2000–2019: A Review of 83 Cases
Source: J Fungi (Basel). 2021 Jun 4;7(6):448. doi: 10.3390/jof7060448 (PMC8229079; doi:10.3390/jof7060448)
Supplement: Supplementary file 1 [file jof-07-00448-s001.zip › jof-1241503-supplementary.pdf]

Table S1. Details of 83 paediatric histoplasmosis cases included in this review.

Ref	Country	Age (yrs)	Sex	Disseminated/Single Organ Disease	Organs Effected	Underlying condition	Signs and Symptoms	Diagnostic Technique	Specimens Used for Diagnosis	Treatment	Outcome
17	US	3m	M	Disseminated	Bone marrow	-	Fever<br>HSM	Histopathology<br>Ag Detection	Bone marrow<br>Serum<br>Urine	AmB + Itra	Improved
18	India	11	M	Disseminated	Lymph-nodes<br>Bone marrow	HIV	Fever<br>Abdominal pain<br>Vomiting	Histopathology<br>Culture	Lymph Node<br>Bone marrow<br>Lymph Node<br>Bone marrow	AmB	Death
19	US	16	M	Disseminated	Lungs<br>Bone marrow	HIV<br>Tx-Kidney	Fever<br>Cough<br>Malaise<br>Vomiting<br>Weight Loss<br>Respiratory distress	Microscopy<br>Ag Detection	BAL<br>Urine<br>Serum	AmB + Itra	Improved
	US	11	F	Disseminated	Lungs	Tx-Kidney	Fever<br>Cough<br>Headache<br>Splenomegaly	Ag Detection	Urine	Itra + Vori	Recurrence (compliance issues)
	US	16	F	Disseminated	Pericard<br>Lungs<br>Bone marrow	Tx-Kidney	Fever<br>Diarrhoea<br>Vomiting<br>Weight loss<br>Respiratory Distress	Ag Detection	Pericardial fluid<br>BAL<br>Urine<br>Serum	AmB + Itra	Lost to Follow Up
	US	11	F	Disseminated	Lungs<br>Bone marrow	Tx-Kidney	Fever<br>Fatigue<br>Cough<br>Abdominal pain<br>Weight loss	Microscopy<br>Ag Detection<br>Ab Detection (CF)	BAL<br>Urine<br>Serum	AmB + Itra	Not specified
20	India	15	M	Disseminated	Lung<br>Lymph nodes<br>Bone marrow	Tx-Kidney	Fever<br>Cough<br>Malaise<br>Abdominal Pain<br>Chest pain<br>Splenomegaly	Histopathology	Lymph Node<br>Bone marrow	AmB+ Itra + Vori	Cure
21	Brazil	13	M	Disseminated	Lung<br>Lymph-nodes	Not specified	Cough<br>Lymphadenopathy	Ag Detection	Serum	Itra	Improved
22	India	13	M	Disseminated	Bone<br>CNS<br>Lymph-nodes	Not Specified	Fever<br>Anorexia<br>Lymphadenopathy<br>HSM<br>Weight loss	Histopathology<br>Culture	Bone (skull + tibia)<br>Bone (skull + tibia)	AmB + Itra	Cure<br>Treatment ongoing

23	US	6m	F	Disseminated	Lung<br>Bone<br>Marrow	HLH	Fever<br>Lethargy<br>HSM<br>Respiratory distress	Histopathology	Bone marrow	AmB	Cure<br><br>Treatment ongoing
								Ag Detection	Serum<br>Urine<br>CSF		
								Ab Detection (ID)	Serum		
24	Nepal	14	M	Disseminated	Lung<br>Skin	Rheumatic Heart Disease	Fever<br>Cough<br>HSM<br>Skin lesions<br>Weight loss	Histopathology	Lung<br>Skin	AmB + Itra	Improved<br><br>Treatment ongoing
25	US	10	M	Disseminated	Lung<br>Skin<br>Bone marrow	HIES (STAT-3 def)	Fever<br>Abdominal pain<br>Skin lesions	Microscopy	BAL	AmB + Posa	Improved<br><br>Treatment ongoing
								Ag Detection	Serum<br>Urine		
	US	13	F	Disseminated	GI	HIES (STAT-3 def)	Abdominal pain<br><br>Weight loss	Histopathology	GI Tissue	AmB + Itra	Improved<br><br>Treatment ongoing
								Culture	GI Tissue		
								Ag Detection	Urine		
26	India	7	F	Disseminated	Bone marrow<br>Lymph nodes<br>Skin<br>Lungs	-	Fever<br>Ostalgia<br>Weight loss<br>HSM<br>Respiratory distress	Histopathology	Lymph Node<br>Bone marrow	AmB	Death
								Culture	Bone marrow<br>Skin<br>Blood		
								Gene Sequencing	Skin		
27	US	13	M	Disseminated	Lymph	Crohn's Disease	Lymphadenopathy	Serology (Ab/Ag not specified)	Not Specified	Itra	Cure
28	Colombia	7	M	Laryngeal	Larynx	Tx-Kidney	Diarrhoea<br>Odynophagia<br>Tonsillitis<br>Anorexia<br>Headache<br>Fever<br>Stridor<br>Lymphadenopathy	Ab Detection (ID)	Serum	AmB + Itra	Cure
								Histopathology	Epiglottis biopsy		
29	India	17	M	Disseminated	Bone<br>Soft Tissue	Previous MTB<br><br>Common Variable Immunodeficiency	Arthralgia<br>Joint swelling<br>Abdominal pain<br>Ascites<br>Fever<br>Weight loss<br>HSM	Microscopy	Joint Aspirate	AmB + Itra	Cure
30	US	6	M	Disseminated	CNS	EBV	Fever<br>Nausea<br>Abdominal pain<br>Headache<br>Phonophobia	Ag Detection	Urine<br>CSF	AmB + Itra	Not Specified
								Ab Detection (CF)	Serum		

							Photophobia				
31	US	8	M	Pulmonary	Lungs	Not Specified	Fever<br>Cough<br>Abdominal pain	Ag Detection<br>Ab Detection (CF)	Serum<br>Serum	Itra	Cure
	US	5	F	Pulmonary	Lungs	Not Specified	Fever<br>Cough<br>Abdominal pain	Ag Detection<br>Ab Detection (CF)	Serum<br>Serum	Itra	Cure
32	Nigeria	13	F	Disseminated	CNS<br>Lungs<br>Lymph nodes	Not Specified	Lymphadenopathy<br>Oliguria<br>Seizures<br>Respiratory distress<br>Ascites	Histopathology	Lymph Node	No treatment	Death
33	US	17	M	Disseminated	Lymph-nodes	EBV	Fatigue<br>Night-sweats<br>Headaches<br>Weight loss<br>Lymphadenopathy	Histopathology	Lymph node	Itra	Not Specified
34	US	6	M	CNS	CNS	-	Headache<br>Reduced mobility<br>Facial droop<br>Slurred speech	Culture<br>Ab Detection (CF)<br>Ag Detection	CSF<br>CSF, Serum<br>CSF, Serum	AmB + Itra	Cure
35	India	5	F	Disseminated	Bone marrow	Not Specified	Abdominal distention<br>Petechiae	Histopathology<br>Serology (not specified)	Bone marrow<br>Not Specified	AmB	Improved
36	US	12	F	Disseminated	Lung<br>Lymph-nodes	Not Specified	Neck and shoulder pain<br>Chest pain<br>SOB	Histopathology<br>Ab Detection (CF+ID)	Lymph node, Lung Tissue<br>Serum	AmB + Itra	Recurrence
37	US	4m	F	Disseminated	Bone marrow	-	Fever<br>HSM	Ag Detection	Urine	AmB + Itra	Not Specified
38	US	17	M	Disseminated	Liver<br>Bone marrow<br>Lymph-nodes	STAT1 mutation	Lymphadenopathy<br>Fever<br>Weight loss	Not specified	Not specified	Not specified	Recurrence
	US	7	F	Disseminated	Lymph-nodes<br>Lung	STAT1 mutation	Fever<br>HSM<br>Lymphadenopathy<br>SOB	Not specified	Not specified	Itra	Recurrence
39	Brazil	13	F	Pulmonary	Lung	Lymphoma	Not specified	Histopathology	Lung	Itra	Improved
		13	M	Pulmonary	Lung	-	Not specified	Histopathology	Lung	Itra	Improved
40	China	11	M	Osseous	Bone	-	Bone/joint swelling<br>Skin nodule	Histopathology	Bone	Debridement<br>Vori	Cure
41	US	17m	M	Disseminated	Lung<br>Bone marrow	T-cell deficiency	Fever	Histopathology	Bone marrow	AmB	Improved<br>Persistently elevated urine

								Microscopy<br>Ag Detection	BAL<br>Urine		Ag
42	Brazil	14	F	Disseminated	Lung<br>Lymph-<br>nodes	Juvenile SLE	Cough<br>Abdominal Pain<br>Fever<br>Diarrhoea<br>Vomiting<br>Impetigo<br>HSM	Histopathology<br>Culture<br>Ab Detection (ID)	Lymph node<br>GI Tissue<br>Lymph node<br>Serum	AmB	Death
43	Guinea-<br>Bissau	12	M	Disseminated	Lung<br>Lymph-<br>nodes	-	Lymphadenopathy<br>with cutaneous<br>fistulas	Culture	Lymph node	AmB + Itra +<br>Posa	Cure
44	India	4	F	Disseminated	GI<br><br>Lymph-<br>nodes	HIES	Fever<br>Abdominal pain<br>Diarrhoea<br>Weight loss<br>Lymphadenopathy<br>HSM	Histopathology	GI Tissue	Antibiotics	Improved
45	US	6m	M	Disseminated	Bone<br>marrow	-	Fever<br>HSM	Histopathology<br>Ag Detection<br>Ab Detection (CF)	Bone Marrow<br>Urine<br>Serum<br>Serum	AmB + Itra	Cure
46	US	13	F	CNS	CNS	-	Confusion<br>Headaches<br>Neck Pain<br>Back Pain<br>Abdominal pain<br>Hearing loss<br>Facial Numbness<br>Slurred Speech<br>Fever<br>Altered mental<br>status	Ag Detection<br>Ab Detection (ID)	Blood<br>CSF<br>Serum	AmB + Itra	Improved<br><br>Treatment<br>ongoing
47	US	8	M	Pulmonary	Lung	Not<br>Specified	Wheeze	Histopathology	Lung tissue	Itra	Not Specified
	US	4	M	Pulmonary	Lung	Wilms<br>tumour<br>Chemothera<br>py	None (incidental<br>finding)	Histopathology	Lung tissue	Antifungal<br>(not specified<br>+ resection	Not Specified
48	US	16	M	Disseminated	Lung<br>Lymph<br>nodes	Idiopathic<br>chronic<br>anterior<br>uveitis	Fever<br>Night sweats<br>Cough<br>Headache<br>Dysphagia<br>Lymphadenopathy<br>Chest Pain	Ab Detection (CF)<br>Ag Detection	Serum<br>Urine	AmB + Itra	Cure
49	Us	12	M	Pulmonary	Lung	-	Cough<br>Fever<br>Chest pain	Histopathology<br>Ab Detection (ID)<br>Ag Detection	Lung Tissue<br>Serum<br>Pleural Fluid	AmB + Itra	Cure

50	Brazil	13	M	Pulmonary	Lung	Not Specified	Fever<br>Headache<br>Myalgia<br>Malaise	Serology (not specified)	Not specified	Not specified	Improved<br>Persistent pulmonary nodule on CT
51	India	3	-	Disseminated	Skin	-	Skin lesions<br>HSM<br>Mouth ulceration	Histopathology	Skin	AmB + Itra	Recurrence
52	US	16	F	Disseminated	Lung<br>Lymph-nodes	Crohn's Disease	Abdominal Pain<br>Fatigue<br>Anorexia<br>Nausea<br>Headache<br>Myalgia<br>Dysuria<br>SOB	Ag Detection	Serum<br>Urine	Itra*	Cure
	US	14	F	Pulmonary	Lung	Crohn's Disease	Fever<br>Headache<br>Myalgia<br>Arthralgia<br>Cough<br>Anorexia<br>Weight loss<br>Dizziness	Serology (not specified)	Not specified	AmB + Itra	Cure
	US	13	M	Pulmonary	Lung	Crohn's Disease	Fever<br>Malaise	Serology (not specified)	Serum<br>Urine	AmB + Itra	Lost to Follow Up
	US	15	F	Disseminated	Lung<br>Lymph-nodes	Crohn's Disease	Fever<br>Abdominal pain<br>Cough<br>Fatigue	Ab Detection (not specified)<br>Ag Detection	Serum<br>Serum<br>Urine	AmB + Itra	Improved<br><br>Persistent urine Ag positive
53	India	16	F	Disseminated	Skin<br>Bone	AIDS	Fever<br>Diarrhoea<br>Skin lesions<br>Lymphadenopathy<br>HSM<br>Weight loss	Histopathology	Bone marrow	AmB	Death
54	US	8	M	Disseminated	Lung<br>Lymph-nodes	Crohn's Disease	Fever<br>Night sweats<br>Cough<br>Anorexia<br>Abdominal pain<br>Diarrhoea<br>HSM	Culture<br>Ag Detection<br>Ab Detection (CF)	Lymph Node<br>Urine<br>Serum	Itra	Cure
55	US (mother from Guatemala	4wk	F	Disseminated	Skin<br>Lung<br><br>CNS	HIV	Fever<br>Skin rash<br>Respiratory distress<br>Seizures	Culture<br>Ag Detection<br>Microscopy	CSF<br>Urine<br>CSF	AmB + Itra	Cure

	– vertical transmission)						Hepatomegaly				
56	US	15	F	Disseminated	Lung<br>Lymph-nodes	GIST<br>Chemotherapy	Fever<br>Cough	Ab Detection (CF+ID)	Serum	No treatment	Cure
57	US	8	F	Unclear	Unclear	Juvenile RA	Not specified	Ab Detection (not specified)<br>Ag Detection	Serum<br>Not specified	AmB + Itra	Cure
		10	F	Unclear	Unclear	Juvenile RA	Fever	Ab Detection (not specified)<br>Ag Detection	Serum<br>Not specified	AmB + Itra	Cure
		17	F	Unclear	Unclear	Juvenile RA	Fever	Ab Detection (not specified)<br>Ag Detection	Serum<br>Not specified	AmB + Itra	Cure
		13	F	Unclear	Unclear	Juvenile RA<br>Psoriasis	Fever	Ab Detection (not specified)<br>Ag Detection	Serum<br>Not specified	AmB + Itra	Cure
		16	M	Unclear	Unclear	Uveitis	Not specified	Ab Detection (not specified)<br>Ag Detection	Serum<br>Not Specified	AmB + Itra	Cure
58	India	6	F	Disseminated	Lung<br>Bone marrow	Not Specified	Fever<br>Cough<br>Abdominal pain<br>Oedema<br>HSM	Histopathology	Bone marrow	Not specified	Not specified
		1	M	Disseminated	Bone marrow	HIV	Fever<br>Night sweats<br>Abdominal distention<br>Oedema<br>Weight loss<br>Anorexia<br>Malaise<br>Myalgia	Histopathology	Bone marrow	Not specified	Not specified
59	US	14	F	Disseminated	Lung<br>GI<br>Lymph-nodes	HIES	Abdominal pain<br>Fever<br>Weight loss<br>Fatigue<br>Cough	Histopathology<br>Ag Detection<br>Ab Detection (CF +ID)	GI Tissue<br>Urine<br>Serum	Itra	Cure
60	US	15	F	Disseminated	Not specified	Sarcoidosis<br>Tx-Kidney	Fever<br>Multi-organ failure	Autopsy	Not specified	No treatment	Death
61	US	5m	M	Pulmonary	Lung	-	SOB	Ab Detection (CF + ID)<br>Ag Detection	Serum<br>Urine	Itra	Cure
62	Brazil	13	M	Disseminated	Skin	AML	Skin nodules	Histopathology	Skin	AmB + Itra + Fluc	Cure
63	US	15	F	Disseminated	Lung	Tx-Kidney	Not specified	Ag Detection	Serum	Itra + Vori	Improved

									Urine		
64	Africa	10	F	Disseminated	Skin<br>Lymph-nodes	-	Skin lesions<br>Lymphadenopathy	Histopathology<br>Ab Detection (ID)	Lymph node<br>Serum	AmB	Improved<br>Residual Skin Lesions
65	Pakistan	5	M	Disseminated	Lymph-nodes	Not Specified	Fever<br>Weight loss	Histopathology	Lymph node	Not specified	Not specified
66	US	8m	F	Disseminated	Lungs<br>Bone marrow	-	Fever<br>Cough<br>HSM	Histopathology<br>Ag Detection<br>Ab Detection (ID)<br>Culture	Bone marrow<br>Urine<br>Serum<br>Blood	AmB + Itra	Cure
67	France (Recent Travel to Africa)	2	F	Disseminated	Skin<br>Bone	HIV	Frontal Bone Swelling<br>Fever<br>Weakness	Histopathology<br>Culture	Skin<br>Bone<br>Skin	AmB + Itra + Fluc	Improved
68	US	7	F	Disseminated	Lung<br>Lymph-nodes	Juvenile RA	Fever<br>Sore throat<br>Weight loss<br>Splenomegaly	Histopathology<br>Ag Detection	Lymph Node<br>Urine	Itra	Improved<br><br>Persistent symptoms
69	US	11	F	Disseminated	Lung<br>Skin<br>Lymph-nodes	HIV	Weight Loss<br>Cough<br>Skin Lesions<br>Lymphadenopathy<br>HSM	Histopathology	Skin	AmB	Death
70	Venezuela	3m	M	Disseminated	Lung<br>Lymph-nodes	Not Specified	Fever<br>SOB<br>Jaundice	Histopathology<br>Culture	Lymph node<br>Lymph Node	No treatment	Death
71	US	3	M	Disseminated	Lung<br>Bone marrow<br>Lymph-nodes	Interferon-gamma receptor-1 Deficiency	Fever<br>HSM<br>Lymphadenopathy	Ab Detection (ID + CF)<br>Culture	Serum<br>BAL<br>Bone marrow	AmB + Keto	Recurrence
72	Africa	17	M	Disseminated	Skin<br>Bone<br>Soft Tissue	Not Specified	Skin ulceration<br>Pharyngeal fistula	Histopathology	Bone<br>Soft tissue	AmB	Cure
73	US	8	M	Disseminated	Lymph-nodes	-	Flu-like illness<br>Vomiting<br>Weight loss	Ab Detection (CF + ID)	Serum	No treatment	Improved<br><br>Oesophageal diverticulum due to extensive lymphadenopathy
	US	10	M	Disseminated	Lymph-nodes	Not Specified	Cough<br>Fatigue<br>Weight loss<br>Chest Pain<br>Dysphagia<br>Lymphadenopathy	Ab Detection (CF)	Serum	Itra	Cure
	US	14	M	Disseminated	Lymph-nodes<br>Lung	-	Dysphagia<br>Night Sweats<br>Cough	Ab Detection (CF+ID)	Serum	Itra	Cure
74	US	17	F	Disseminated	Lung<br>Bone	Juvenile RA	Cough<br>Fever	Ag Detection	Urine	AmB + Itra	Cure

					marrow		Nausea<br>Vomiting<br>SOB	Ab Detection (ID + CF)	Serum		
75	US	5m	F	Disseminated	Bone marrow	-	Fever<br>Diarrhoea<br>Rhinorrhoea<br>Abdominal distention<br>HSM<br>Irritability	Histopathology	Bone Marrow	Not specified	Not specified
76	US	11	F	Disseminated	Lymph-nodes	-	Neck pain<br>Lymphadenopathy	Histopathology<br>Ab Detection (CF+ID)	Lymph Node<br>Serum	Itra	Cure
77	Paraguay	11m	F	Disseminated	Bone<br>Lungs	-	Fever<br>Cough<br>Respiratory Distress<br>Anorexia<br>Weight loss<br>Arthralgia<br>Joint swelling<br>Periorbital swelling	Histopathology	Joint tissue	Itra	Improved
78	Africa	3	M	Disseminated	Skin<br>Lymph-nodes<br>Bone marrow	HIV	Skin rash<br>Lymphadenopathy<br>HSM	Histopathology	Not specified	AmB + Itra	Lost to Follow Up
79	Panama	14	F	Disseminated	Bone marrow<br>CNS<br>Liver<br>Spleen<br>Pancreas	ALL<br>HIV	"Sepsis-like-picture"	Culture	Blood<br>Bone marrow	AmB + Itra	Improved
80	US	6	F	Disseminated	Bone<br>Ocular	ALL<br>Chemotherapy	Fever<br>Malaise<br>HSM<br>Choroidal lesions on fundi	Histopathology<br>Ag Detection<br>Culture	Bone Marrow<br>Urine<br>Bone Marrow<br>Blood	AmB	Cure
81	US	3	F	Pulmonary	Lungs	-	Fever<br>SOB<br>Malaise<br>Weight loss<br>Abdominal pain	Ab Detection (ID +CF)	Serum	No treatment	Cure

F=female, M=male, SOB = shortness of breath, Itra = itraconazole, Keto = ketoconazole, AmB = Amphotericin B, Vori = voriconazole, ID = immunodiffusion, CF = complement fixation, BAL = Broncho-alveolar lavage, d = day, m = month(s), HIV = Human Immunodeficiency Virus, Ab = Histoplasma antibody, Ag = Histoplasma antigen, ALL = Acute Lymphoblastic Leukaemia, RA = Rheumatoid Arthritis, AML = Acute Myeloid Leukaemia, GIST = Gastrointestinal stromal tumour, Tx = transplant, HIES = hyper-immunoglobulin E syndrome, SLE = systemic lupus erythematosus, EBV = Epstein Barr Virus infection, MTB = Mycobacterium tuberculosis infection, HLH = hemophagocytic lymphohistiocytosis, HSM = hepatosplenomegaly.
